# Pharmacokinetics of the Perioperative Use of Cancer Chemotherapy in Peritoneal Surface Malignancy Patients

**DOI:** 10.1155/2012/378064

**Published:** 2012-06-13

**Authors:** K. Van der Speeten, K. Govaerts, O. A. Stuart, P. H. Sugarbaker

**Affiliations:** ^1^Department of Surgical Oncology, Oost-Limburg Hospital, Schiepse Bos 6, 3600 Genk, Belgium; ^2^Department of Biochemistry, Faculty of Medicine, University Hasselt, 3590 Diepenbeek, Belgium; ^3^Washington Cancer Institute, Washington Hospital Center, Washington, DC 20010, USA

## Abstract

*Background*. The peritoneal surface is an acknowledged locoregional failure site of abdominal malignancies. Previous treatment attempts with medical therapy alone did not result in long-term survival. During the last two decades, new treatment protocols combining cytoreductive surgery with perioperative intraperitoneal and intravenous cancer chemotherapy have demonstrated very encouraging clinical results. This paper aims to clarify the pharmacologic base underlying these treatment regimens. *Materials and Methods*. A review of the current pharmacologic data regarding these perioperative chemotherapy protocols was undertaken. *Conclusions*. There is a clear pharmacokinetic and pharmacodynamic rationale for perioperative intraperitoneal and intravenous cancer chemotherapy in peritoneal surface malignancy patients.

## 1. Introduction

The peritoneal surface is an established failure site for digestive and gynecological malignancies as well as the primary location for some tumors [1–7]. Historical attempts at cure with medical therapy alone have never resulted in long-term survival. During the last two decades, new treatment modalities combining extensive cytoreductive surgery (CRS) and perioperative intraperitoneal and intravenous cancer chemotherapy have emerged. In several phase II and phase III trials, these new therapeutic approaches for peritoneal surface malignancy have shown very promising clinical results [8–18]. Although further clinical trials are mandatory, another route of exploration is equally important for further improvement of these combined treatment regimens. Pharmacologic studies of perioperative cancer chemotherapy should guide further progress in this field and offer clues for a more standardization. This paper aims to review the current pharmacologic data and point out areas of controversy needing clarification.

## 2. Dose Intensification

Dose intensification between the peritoneal compartment and the body compartment is the basic underlying pharmacologic rationale for all intraperitoneal therapy as initially stated by Dedrick et al. [19, 20]. The two above-mentioned compartments are separated by a semi permeable membrane the peritoneum. In 1941, Baron reported an elaborated description of the ultrastructure of the peritoneum in man [21]. The peritoneum consists of a monolayer of mesothelial cells supported by a basement membrane and five layers of connective tissue which account for a total thickness of 90 *μ*m. The connective tissue layers include interstitial cells and a matrix of collagen, hyaluronan, and proteoglycans. The cellular component consists of fibroblasts, pericytes, parenchymal cells, and blood capillaries. Contrary to intuitive thinking, it is not the mesothelial lining which is the main transport barrier. Flessner et al. demonstrated in a rodent model that neither removal of the stagnant fluid layer on the mesothelium nor removal of the mesothelial lining influenced the transport over the barrier [22]. This has been confirmed in human studies in patients undergoing partial or total peritonectomy showing that the clearance of mitomycin C was not significantly changed by the removal of the mesothelium [23, 24]. Basic research suggests that rather the blood capillary wall and the surrounding interstitium are the most important barriers for transport from the peritoneal space to the plasma [25]. Fluid enters the vascular compartment by diffusion from the peritoneal compartment or by absorption through the peritoneal lymphatic stomata which are concentrated on the diaphragmatic surface [26, 27]. Diffusion of fluid through the parietal peritoneum generally results in flow to the plasma compartment. Drainage through the visceral peritoneum covering the surfaces of liver, spleen, stomach, small and large bowel, and mesentery is into the portal venous blood [28].

**Figure 1:**
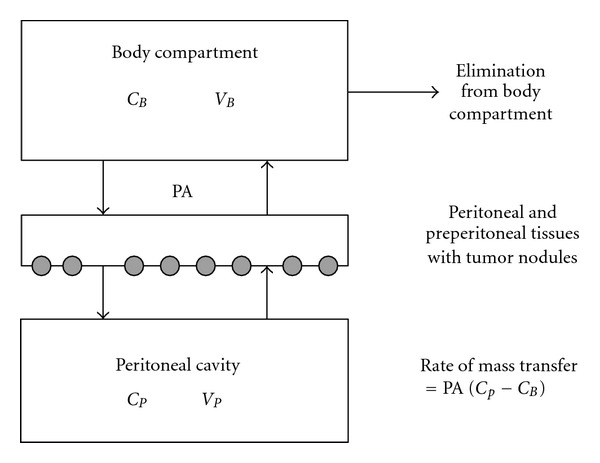
Traditional two-compartment model of peritoneal transport in which transfer of a drug from the peritoneal cavity to the blood occurs across the “peritoneal membrane.” The permeability-area product (PA) governs this transfer and can be calculated by measuring the rate of drug disappearance from the cavity and dividing by the overall concentration difference between the peritoneal cavity and the blood (or plasma). *C*_*B*_: the free drug concentration in the blood (or plasma); *V*_*B*_: volume of distribution of the drug in the body; *C*_*P*_: the free drug concentration in the peritoneal fluid; *V*_*P*_: volume of the peritoneal cavity. Modified from R. L. Dedrick, M. F. Flessner: pharmacokinetic problems in peritoneal drug administration: Tissue penetration and surface exposure [31].

The two-compartment Dedrick model of intraperitoneal chemotherapy is shown in Figure 1. A simplified mathematical formula describes the transport as follows: rate of mass transfer = PA (*C*_*P*_ − *C*_*B*_), where PA = permeability area (PA = effective contact area *x* permeability), *C*_*P*_ = concentration in peritoneal cavity, and *C*_*B*_ = concentration in the blood [29]. This formula indicates the importance of the size of the effective contact area of the peritoneal membrane. One should keep in mind that although the equation permits calculation of the pharmacokinetic advantage, the model does not predict the actual penetration of the cancer chemotherapy drug into the tissue or tumor nodule [30]. It neither predicts the value of the effective contact area. It simply describes the transfer between two compartments.

## 3. Drugs Used in Perioperative Cancer Chemotherapy Protocols

Table 1 provides an overview of drugs commonly used in perioperative cancer chemotherapy protocols and their main pharmacologic characteristics.

### 3.1. Mitomycin C

Mitomycin C is an alkylating antibiotic whose most important mechanism of action is through DNA cross-linking. Although mitomycin C is not regarded as a prodrug, it is not active against cancerous tissue as the unchanged molecule. The drug is modified as it enters the cell into an active state [34]. It has been used extensively in intraperitoneal cancer chemotherapy treatment protocols in appendiceal, gastric, and colorectal peritoneal carcinomatosis (PC) patients [8, 24, 35, 36]. Barlogie et al. suggested in vitro thermal enhancement of mitomycin C [37]. Controversies still exist regarding the proper dosimetry of the chemotherapy solution. Some institutions use a single dose of mitomycin C, others a double dose, and still others triple dose the drug over a 90-minute time period [38–40]. A remarkable difference in drug dosimetry between different groups of investigators is reported. Van Ruth and coworkers at the Dutch Cancer Institute reported a dose-finding study [40]. Their data suggest that a dose of 35 mg/m² resulted in the highest peritoneal/plasma area under the curve (AUC) ratio with acceptable toxicity. In order to maintain the concentration throughout the 90 minutes perfusion time, the dose was divided into three fractions: 50% at the start, 25% after 30 minutes, and 25% at 60 minutes. The toxicity profile of mitomycin C, including anastomotic dehiscence and impaired wound healing, has been well characterized [24, 41–43]. Our data suggest large amounts of mitomycin C (62%) remain within the body compartment after the 90-minute hyperthermic intraperitoneal treatment [24].

### 3.2. Cisplatin

Cisplatin (*cis*-diamminedichloroplatinum-III CDDP) causes apoptotic cell death by formation of DNA adducts [44]. It has been well studied in the setting of adjuvant intraperitoneal chemotherapy of residual small volume ovarian cancer after CRS. Three randomized trials showed a significant survival benefit [45–47]. In the setting of cytoreductive surgery and hyperthermic intraperitoneal peroperative chemotherapy (HIPEC), cisplatin has been used for intracavitary therapy of ovarian cancer, gastric cancer, and peritoneal mesothelioma. Urano and coworkers showed an excellent in vitro and in vivo thermal augmentation of cisplatin [48].

### 3.3. Oxaliplatin

Oxaliplatin (oxalato-1,2-diaminocyclohexane-platinum(II)) is a third generation platinum complex with a similar cytotoxic mechanism as cisplatinum. In contrast with cisplatin, it has a proven activity in colorectal and appendiceal malignancies [49]. Its clinical use in PC patients as a component of bidirectional intraoperative chemotherapy has been pioneered by Elias and Sideris [50]. In a dose escalation and pharmacokinetic study, they showed that 460 mg/m² of oxaliplatin in 2 L/m² of chemotherapy solution over 30 minutes was well tolerated [51]. The low AUC ratio is compensated by the rapid absorption of the drug into the tissue. In contrast to cisplatin and mitomycin, oxaliplatin is not stable in chloride-containing solutions and can only be administered in dextrose 5% [52]. This may result in serious electrolyte disturbances and hyperglycemia during the intracavitary therapy [53].

A recent murine pharmacokinetic study with oxaliplatin confirmed its substantial heath augmentation [54].

### 3.4. Carboplatin

Carboplatin ((1,1-cyclobutanedicarboxylate)platinum(II)) is a higher molecular weight platinum compound than cisplatin which at the present time is mostly used in normothermic intraperitoneal chemotherapy protocols in patients with advanced ovarian cancer. Cjezka et al. in a clinical study with normothermic carboplatin reported a relative bioavailability (calculated as AUC values) which was at least 6-times higher in the intraperitoneal fluid than in the serum for 48 hours [55]. Los and coworkers compared carboplatin and cisplatin after intraperitoneal administration in a rat model of peritoneal carcinomatosis [56]. Their data demonstrate that despite a clear pharmacokinetic advantage of carboplatin over cisplatin, its capacity to penetrate into peritoneal cancer nodules and tumor cells is far lower than that of cisplatin. These data limit its clinical application.

### 3.5. Doxorubicin

Doxorubicin (C_27_H_29_NO_11_) or hydroxyldaunorubicin (adriamycin) is an anthracycline antibiotic. Although being categorized as a DNA-intercalating drug, the actual mechanism of action is a critical interaction of doxorubicin with the cell surface membrane [57, 58]. Because of its wide in vitro and in vivo activity against a broad range of malignancies, its slow clearance from the peritoneal compartment due to the high molecular weight of the hydrochloride salt (579, 99 Dalton), its favorable area under the curve ratio of intraperitoneal to intravenous concentration times of 230, and the absence of risk for dose-limiting cardiotoxicity when used as a single-shot intraperitoneal instillation, doxorubicin was considered a potential beneficial agent for perioperative intraperitoneal delivery. This was supported by both experimental and clinical pharmacokinetic data [59–64].

### 3.6. Gemcitabine

Gemcitabine (2′,2′-difluorodeoxycitidine) is a pyrimidine analogue with a wide range of in vitro cytotoxic activity, particularly against pancreatic cancer. Pestiau et al. investigated the pharmacokinetics and tissue distribution of intraperitoneal gemcitabine in a rat model [65]. The AUC ratio (intraperitoneal/intravenous) after intraperitoneal administration was 26.8 ± 5.8 and as such favorable for intraperitoneal administration. Several investigators explored the use of normothermic intraperitoneal gemcitabine in advanced cancer outside the setting of cytoreductive surgery [66–68]. Resected advanced pancreatic cancer with high risk of recurrence in the operative field is a potential indication for intraoperative intraperitoneal administration of heated gemcitabine in an adjuvant setting [69].

### 3.7. Melphalan

Melphalan (L-phenylalanine mustard) is a chemotherapy drug belonging to the class of nitrogen mustard alkylating agents. Alberts et al. were the first to investigate the pharmacokinetics of intraperitoneal melphalan [70]. Melphalan systemic absorption from the peritoneal cavity averaged only 39% of the administered dose. Urano showed a remarkable heat augmentation of melphalan [48]. Glehen and coworkers investigated the effect of hyperthermia on the pharmacokinetics of intraperitoneal melphalan in a rat model [71]. Hyperthermia decreased the AUC of peritoneal fluid without increasing the plasma AUC. Intra-abdominal tissue concentrations were markedly elevated compared to normothermic controls. Sugarbaker et al. in a pharmacokinetic and phase-II study of intraoperative intraperitoneal melphalan showed that 90% of the cancer chemotherapy drug was absorbed during the 90-minute procedure with a 30-times higher exposure at the peritoneal surface than in the blood [72]. Concentrations in tumor nodules were 10-times higher than concentrations in the blood. This favorable pharmacokinetic profile and tissue distributions, combined with cytotoxic activity against a wide range of malignancies, makes melphalan an excellent salvage drug for intraperitoneal treatment protocols.

### 3.8. Taxanes

Paclitaxel and docetaxel are taxanes considered for i.p. chemotherapy. The taxanes stabilize the microtubule against depolymerization, thereby disrupting normal microtubule dynamics [73]. They exert cytotoxic activity against a broad range of tumors. Due to their high molecular weight these molecules have a remarkable high AUC ratio of 853 and 861 respectively, [74]. This translates itself into a clear pharmacokinetic advantage for intraperitoneal administration [75]. The data regarding possible thermal augmentation of taxanes are conflicting [76–79]. Taxanes have been used in a neoadjuvant intraperitoneal setting as well as intraoperatively and postoperatively. Postoperative intraperitoneal paclitaxel conferred a survival benefit in this postoperative setting. Their cell-cycle specific mechanism of action makes them a particular good candidate for repetitive application such as in early postoperative intraperitoneal chemotherapy (EPIC) or normothermic adjuvant postoperative intraperitoneal chemotherapy [45, 46, 80–82].

### 3.9. 5-Fluorouracil

5-Fluorouracil is an inhibitor of thymidylate synthase. Since thymidine is the only nucleotide precursor specific to DNA, thymidilate synthase is an obvious target for cytotoxic agents. 5-Fluorouracil is intracellularly metabolized in two steps to its active metabolite, 5-fluoro-2′-deoxyuridine monophosphate (FdUMP). This molecule will, in the presence of reduced folate, bind at the same site and with the same affinity as deoxyuridine monophosphate (dUMP) and ultimately impair the enzymatic activity of the thymidilate synthetase [83]. The action of 5-fluorouracil is therefore cell cycle specific. Also 5-FU by its metabolites 5-fluoro-uridine diphosphate and 5-fluoro-uridine triphosphate gets incorporated in RNA, resulting in a second cytotoxic pathway. Minor augmentation of 5-fluorouracil by mild hyperthermia is reported [84, 85]. 5-Fluorouracil is not chemically compatible with other drugs in a mixed solution for infusion or instillation. These characteristics limit the use of 5-fluorouracil perioperatively to either early postoperative intraperitoneal chemotherapy or intraoperative intravenous 5-fluorouracil.

### 3.10. Pemetrexed

Pemetrexed is a multitargeted antifolate. It is an analogue of folic acid with cytotoxic activity against a variety of malignancies, especially mesothelioma and colon cancer. It belongs to the antimetabolites. It acts mainly as a thymidilate synthase inhibitor but is also unique in terms of cellular transport and lipid solubility [86]. Pestieau et al. reported favorable intraperitoneal pharmacokinetics [87]. It is currently under investigation for the intraperitoneal treatment of peritoneal mesothelioma.

### 3.11. Ifosfamide

Ifosfamide is a prodrug which needs the cytochrome P 450 system of liver or red blood cells to be activated to its active metabolite 4-hydroxyifosfamide. Consequently, it requires intravenous administration rather than intraperitoneal instillation for its cytotoxic activity. It is one of four drugs that show true heat synergy, with 5- to 10-times the duration of tumor control with 41.5°C heat as compared to normal temperatures [48]. It may be an ideal systemic drug to increase the cytotoxicity of hyperthermic intraperitoneal peroperative chemotherapy. Our pharmacokinetic data show the presence of ifosfamide and its active metabolite in peritoneal tumor nodules after intravenous continuous infusion during bidirectional intraoperative chemotherapy. In these bidirectional treatment protocols, intravenous and intraperitoneal routes of administration are combined after CRS inside the operating room.

## 4. Pharmacologic Variables in Perioperative Chemotherapy

Pharmacokinetics describe what the body does to the drug, whereas pharmacodynamics describe what the drug does to the body.  Table 2 summarizes the pharmacokinetic and pharmacodynamic variables involved in perioperative intraperitoneal and intravenous chemotherapy. One of the most challenging problems hindering a further wide application of these new treatment modalities is the compelling variety of regimens available worldwide. These protocols are sometimes based on little or no pharmacologic data. Furthermore this variability in dosimetry and technology makes multicenter registry or trials very difficult. The international scientific community must come up with a consensus on standardizing the application. This should be based on a thorough review of the available pharmacologic data and clinical results.

## 5. Pharmacologic Controversies 

### 5.1. Concentration-Based or Body Surface Area-(BSA-) Based Dosimetry

Most groups use a drug dose based on calculated body surface area (mg/m^2^). However, Rubin et al. demonstrate that there is an imperfect correlation between actual peritoneal surface area and calculated body surface area and there may be sex differences in peritoneal surface areas, which in turn affects absorption characteristics [88]. The female has a 10% larger peritoneal surface in proportion to body size than the male. There have been attempts to estimate the functional peritoneal surface area through applying stereological methods to computer tomography (CT) scans by extrapolating data from cadaver measurements [89, 90]. Body surface area is an accurate predictor of drug metabolism and is useful for estimating systemic drug toxicity. 

Some groups use a totally different dosimetry regimen based on concentration. The total amount of cancer chemotherapy is mixed in a large volume of carrier solution (usually six liters) that is placed in a reservoir. For example, Deraco and Rossi at the Milan Cancer Institute use doxorubicin 15.25 mg/m^2^/L and cisplatin 43 mg/m^2^/L with a total volume of 6 liters. Glehen and Gilly from Lyon have used mitomycin C 0.5 mg/kg and cisplatin 0.7 mg/kg in a total volume of 4 to 6 liters [91–94]. In this closed method, the amount of chemotherapy solution in contact with the peritoneal surface is determined by multiple variables: the amount of distention (between 2 and 6 liters) of the abdominal cavity, which is induced by the chemotherapy solution, the patient's sex, the amount of ascites present preoperatively, and the extent of the visceral resection. The big advantage of a concentration-based system is that the residual tumor nodules after CRS are exposed to a constant diffusional force and thus cytotoxicity. Unfortunately, the prize to be paid for a better prediction of the efficacy of the intraperitoneal chemotherapy is a high unpredictability of the plasmatic cancer chemotherapy levels and thus toxicity. Indeed, according to the above-mentioned Dedrick formula of transport over the peritoneal membrane, an increase in the volume of intraperitoneal chemotherapy solution will cause an increase in both diffusion surface and the amount of drug transferred from peritoneal space to plasma. For example, in 10 patients dialyzed with different volumes ranging from 0.5 up to 3 liters, there is a linear rise in mass transfer [95].

Other factors contribute to the controversy over the proper dosage of chemotherapy solution. Some institutions use a single dose of the intraperitoneal drug; others use a double, or even triple, dose of the same drug over a 90-minute period [96–98].

### 5.2. Pharmacokinetics versus Pharmacodynamics

**Figure 2:**
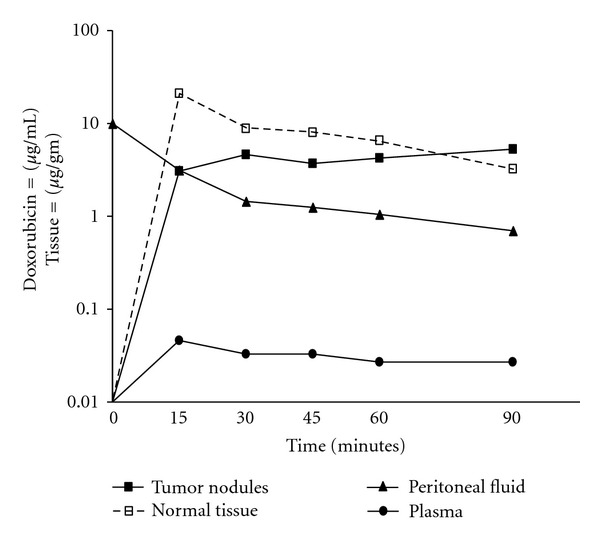
Doxorubicin concentration in plasma, peritoneal fluid, tumor nodules, and normal adjacent tissues [32].

**Figure 3:**
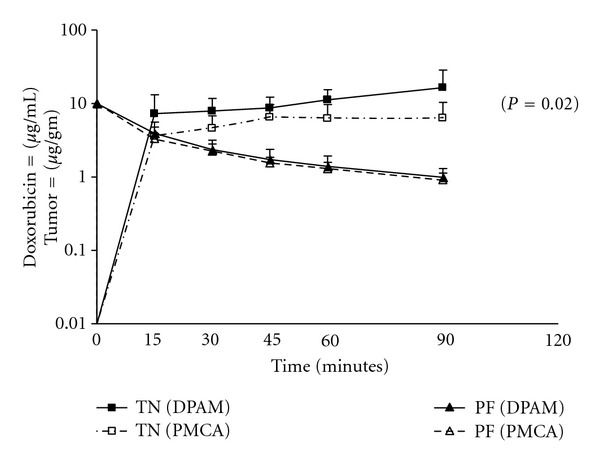
Doxorubicin levels in appendiceal tumor tissue showing diffuse peritoneal adenomucinosis (DPAM) versus peritoneal mucinous carcinomatosis (PMCA). Peritoneal fluid concentrations are also shown. TN: tumor nodule; PF: peritoneal fluid [32].

Until recently, the pharmacologic efficacy of intraperitoneal cancer chemotherapy protocols was assessed by looking at the pharmacokinetics of the i.p. and i.v. compartments. The efficacy of the IP protocol was then quantified by calculating the area-under-the-curve (AUC) ratio of the IP exposure over the AUC of the IV exposure. This, however, does not take into account any pharmacodynamic variables.  Figure 2 demonstrates that the pharmacodynamic event of doxorubicin binding to the tumor nodule results in higher intratumoral concentrations than can be predicted by the simple IP/IV pharmacokinetics [32]. Another example of the equal importance of pharmacodynamics is shown in Figure 3. With identical pharmacokinetics the amount of doxorubicin showing up in the less dense diffuse peritoneal adenomysis (DPAM) subtype of appendiceal malignancy PC is statistically significantly lower than in the more dense peritoneal mucinous carcinomatosis (PMCA) nodules [32]. The identical pharmacokinetic advantage (expressed as AUC IP/IV ratios) resulted in different drug levels according to the density of the tumor nodules; this stressed the importance of pharmacodynamic variables such as tumor nodule density, size, and vascularity. Increased awareness of the pharmacodynamic aspects of these treatment protocols has also been reported by Ceelen et al. [99]. Therefore, it was proposed that the tumor nodule was a more appropriate pharmacological endpoint than AUC ratios.

### 5.3. Adding Intravenous Intraoperative Chemotherapy to the Equation

**Figure 4:**
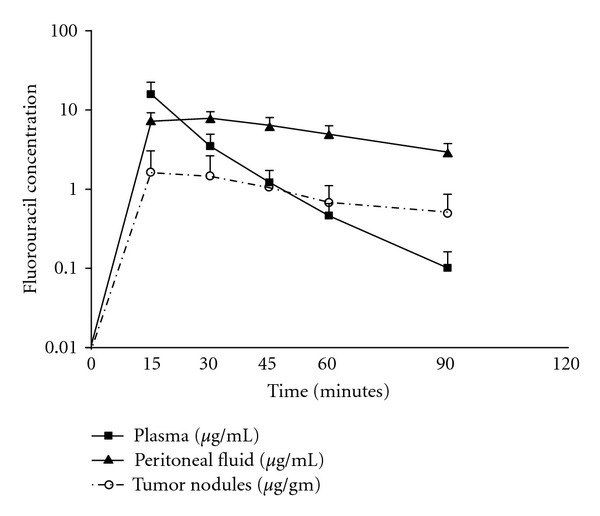
5-Fluorouracil concentrations in plasma, peritoneal fluid, and tumor nodules after intravenous administration during hyperthermic intraperitoneal chemotherapy procedure [33].

By combining intraoperative intravenous and intraoperative intraperitoneal cancer chemotherapy, a bidirectional diffusion gradient is created through the intermediate tissue layer which contains the cancer nodules. This offers opportunities for optimizing cancer chemotherapy delivery to the target peritoneal tumor nodules. In 2002, Elias et al. first reported the clinical use of intraoperative intravenous 5-fluorouracil and leucovorin in conjunction with oxaliplatin-based hyperthermic intraperitoneal perioperative chemotherapy [100].  Figure 4 demonstrates the concentrations of 5-fluorouracil in tumor nodules that were harvested during bidirectional (intraperitoneal doxorubicin and mitomycin C plus rapid infusion intravenous 5-fluorouracil) intraoperative chemotherapy treatment [33]. The rapid distribution of the 5-fluorouracil after IV administration affects all compartments similarly. The metabolism of the 5-fluorouracil on the other hand is mainly restricted to the plasma compartment by the liver. The high level of 5-fluorouracil persists within the peritoneal fluid because the drug can only leave the peritoneal space by back diffusion through the peritoneal and subperitoneal tissues; the enzyme dihydropyrimidine dehydrogenase is not present in the artificial ascites fluid. These data show clear pharmacokinetic advantage for the intraoperative intravenous administration of 5-fluorouracil. Although 5-fluorouracil is administered as a normothermic intravenous solution, it penetrates into the heated tumor nodules. Normothermic administered 5-fluorouracil becomes subject to augmentation of mild hyperthermia of the subperitoneal compartment. Therefore, heat targeting is achieved by modulating the timing of intravenous chemotherapy. 

Recently, we were able to demonstrate a similar pharmacokinetic advantage and heat targeting of intraoperative intravenous ifosfamide (continuous infusion over 90 minutes) [101].

## 6. Conclusions

The last two decades saw the emergence of perioperative cancer chemotherapy protocols in the treatment of PC patients. This has resulted in remarkable clinical successes in contrast with prior failures. Now that the concept is proven, time has come to further improve the treatment protocols. Building more pharmacologic data on perioperative chemotherapy in PC patients should result in both more standardization and better clinical outcome.

## Figures and Tables

**Table 1:** Molecular weight and area under the curve ratios of intraperitoneal exposure to systemic exposure of chemotherapeutic agents used to treat peritoneal carcinomatosis.

Drug	Molecular weight (Daltons)	Area under the curve ratio
5-Fluorouracil	130.08	250
Carboplatin	371.25	10
Cisplatin	300.1	7.8
Docetaxel	861.9	552
Doxorubicin	579.99	230
Etoposide	588.58	65
Floxuridine	246.2	75
Gemcitabine	299.5	500
Irinotecan	677.19	N/A
Melphalan	305.2	93
Mitomycin C	334.3	23.5
Mitoxantrone	517.41	115–255
Oxaliplatin	397.3	16
Paclitaxel	853.9	1000
Pemetrexed	597.49	40.8

**Table 2:** Pharmacokinetic and pharmacodynamic variables involved in the administration of perioperative cancer chemotherapy in peritoneal surface malignancy patients.

Pharmacokinetic variables	Pharmacodynamic variables
Dose	Temperature
Volume	Nodule size of residual tumor
Duration	Density
Carrier solution	Binding
Pressure	Interstitial fluid pressure
Vasoactive agents	Charge
Macromolecular vehicles	Vascularity
